# 

**Published:** 1908-07

**Authors:** 


PUBLISHED MONTHLY. SUBSCRIPTION $1.00

### ATLANTA JOURNAL-RECORD OF MEDICINE

Indexed S. G. O.

Successor to { Atlanta Medical and Surgical Journal, Established 1855, and Southern Medical Record, Established 1870. } 53d Year

Vol. X. ATLANTA, GA., JULY, 1908. No. 4

